# Biallelic mutation of Protocadherin-21 (*PCDH21*) causes retinal degeneration in humans

**Published:** 2010-01-15

**Authors:** Robert H. Henderson, Zheng Li, Mai M. Abd El Aziz, Donna S. Mackay, Mohammad A. Eljinini, Marwan Zeidan, Anthony T. Moore, Shomi S. Bhattacharya, Andrew R. Webster

**Affiliations:** 1 Moorfields Eye Hospital, London, UK; 2 Institute of Ophthalmology, University College of London, London, UK; 3 Alisra Private University, Amman, Jordan; 4 Jordan Hospital, Amman, Jordan

## Abstract

**Purpose:**

To describe the clinical findings and mutations in affected members of two families with an autosomal recessive retinal dystrophy associated with mutations in the protocadherin-21 *(PCDH21)* gene.

**Methods:**

A full genome scan of members of two consanguineous families segregating an autosomal recessive retinal dystrophy was performed and regions identical by descent identified. Positional candidate genes were identified and sequenced. All patients had a detailed ophthalmic examination, including electroretinography and retinal imaging.

**Results:**

Affected members of both families showed identical homozygosity for an overlapping region of chromosome 10q. Sequencing of a candidate gene, *PCDH21*, showed two separate homozygous single-base deletions, c.337delG (p.G113AfsX1) and c.1459delG (p.G487GfsX20), which were not detected in 282 control chromosomes. Affected members of the two families first reported nyctalopia in late teenage years and retained good central vision until their late 30s. No color vision was detected in any proband. The fundus appearance included the later development of characteristic circular patches of pigment epithelial atrophy at the macula and in the peripheral retina.

**Conclusions:**

Biallelic mutations in the photoreceptor-specific gene *PCDH21* cause recessive retinal degeneration in humans.

## Introduction

Retinitis pigmentosa (RP) is a genetically determined disorder in which there is progressive dysfunction and degeneration of the rod and cone photoreceptors. Inheritance may be autosomal dominant, autosomal recessive, and X-linked recessive, and even among these genetic subtypes there is considerable genetic heterogeneity. Most progress has been made in identifying genes causing autosomal dominant and X-linked disease (RetNet). In recessive RP, current genes account for only 50% of families in which retinal disease is the only manifestation [1].

As part of a larger study investigating the genetic causes of RP and early onset retinal dystrophies, we used autozygosity mapping in two consanguineous families with autosomal recessive Retinitis Pigmentosa (arRP)to identify a common autozygous region on chromosome 10q. We subsequently identified null mutations in protocadherin-21 *(PCDH21)*, a photoreceptor-specific gene [2,3], as the cause of the retinal disease in these families.

## Methods

### Recruitment of families

**Figure 1:**
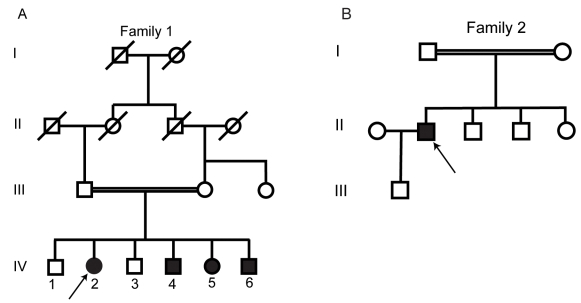
The pedigrees of the two families that took part in the study (denoted family 1 and 2 in the text) are displayed. Open and closed symbols denote unaffected and affected individuals, respectively. Deceased family members are denoted by diagonal lines; arrows indicate the probands in each family. A horizontal double line between parents indicates consanguinity. Panel **A** is a pedigree drawing of family 1, a first cousin consanguineous middle-eastern family, with four affected siblings. Panel **B** shows the nuclear family who took part in the study, and is part of a larger consanguineous pedigree not displayed or available for the study; the parents illustrated are first cousins.

Two families with an adult onset form of arRP were ascertained at our institution and pedigrees constructed (Figure 1). The first (family 1) was a two-generation consanguineous family of Middle-Eastern descent with 4 affected (2 male and 2 female) and two unaffected siblings. The affected individuals ranged in age from 32 to 42 years. The second (family 2) was a consanguineous family of South Asian descent with one affected male individual and three unaffected siblings. Informed consent was obtained from all study participants, and the study conformed to the tenets of the Declaration of Helsinki. Two 10 ml EDTA tubes of peripheral venous blood were drawn from all available family members, including both affected and unaffected individuals, and samples were frozen. DNA was extracted using the Nucleon™ BACC-2 genomic DNA kit (GE Healthcare Life Sciences, Buckinghamshire, UK). Following cell lysis, deproteinization was performed using sodium perchlorate. DNA extraction was achieved with chloroform and Nucleon™ resin before DNA recovery and washing.

### DNA analysis

**Figure 2:**
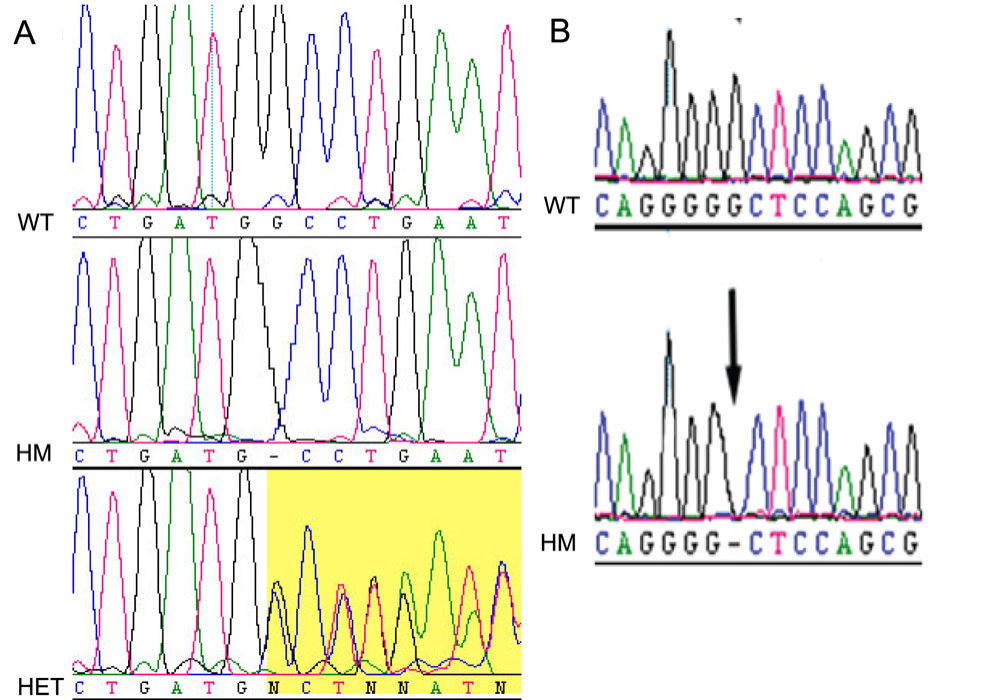
Electropherograms of the mutations detected in Protocadherin-21 (*PCDH21*). Panel **A** illustrates the index c.338delG mutation in family 1; the wild-type (WT) sequence is displayed on the top row. The middle row shows the proband in family 1 (IV-2) with a homozygous (HM) c.338delG change, illustrated with a dash for the missing nucleotide when aligned with the wildtype sequence. The bottom row displays an unaffected parent of family 1 (III-3) with the c.338delG change in the heterozygous state (Het): the latter section of the heterozygous electropherogram shows two superimposed sequences due to the synchronous addition of nucleotides due to two distinct DNA templates derived from the wild type and the shorter mutant alleles of the heterozygote. Panel **B** illustrates the second mutation that was identified in *PCDH21*, in family 2. The top row displays the control individual with the wildtype (WT) allele; while the bottom row displays the affected proband (II-1) with a homozygous (HM) c.1463delG variant.

Genome-wide linkage scans were performed to identify regions of autozygosity (where both alleles are identical by descent and are copies of a common ancestral gene). DNA samples from all affected family members were genotyped using the Affymetrix human GeneChip® Mapping Array (version Xba142 2.0) for family 1 and the GeneChip® Human Mapping 100 K set array for family 2 (Affymetrix, Santa Clara, CA). The detailed methodology for genotyping using the GeneChip® array has been previously described [4]. Briefly, 250 ng of genomic DNA was digested with XbaI (New England Biolabs, Ipswich, MA) for 2 h at 37 °C, ligated with XbaI adaptors using T4 DNA ligase (New England Biolabs). The ligation reaction was diluted in 1:4 (vol/vol) with molecular grade water (Sigma-Aldrich, St. Louis, MO) to 100 µl. Ten µl of the diluted ligation mix was used to setup selection by PCR (Fragment Selection by PCR) in triplicates. The pooled PCR products were purified using QIAGEN MinElute 96 UF plate (Qiagen, Duesseldorf, Germany). The concentration of PCR products was quantified using ND-1000 Nanodrop spectrometry (Thermo Fisher Scientific, Waltham, MA). Purified PCR product (90 ng) was fragmented with 0.25 units DNaseI ('Fragmentation Reagent'; Affymetrix), labeled with 'Labeling Regent' (Affymetrix). The labeling reaction (70 µl) was mixed with 190 μl hybridization reagent and denatured at 99 °C for 10 min (all as detailed in the Affymetrix GeneChip Mapping 100K Assay Manual). Finally the denatured hybridization mixture was injected into Affymetrix Human Mapping XbaI chips and incubated at 48 °C for 16 h, followed by automatic washing and staining in a Fluidics Station 450, and scanned by using the GeneChip® Scanner 3000 7G (Affymetrix). Primers for direct sequencing were as described by Bolz et al. [5]. PCR amplification was performed using a 20-µl reaction mix, including Abgene Reddymix with or without dye (AB795, AB793; Abgene, Epsom, UK). Ten microliters of Reddymix was combined with 5 µl of deionized water, 2 µl (10 pmol) of forward and reverse primer, and 1 µl (50–100 ng) of genomic DNA. All exons were optimized and amplified at 55 °C. DNA fragments were purified using Montage PCR cleanup plates, according to the standard protocol (Millipore, Watford, UK). PCR cleanup (purple plates) was performed using 15 μl of PCR product made up to 100 μl with deionized water (ddH_2_O) and transferred to the purple plates. Ten min of vacuum was applied to the plates until the wells were empty. Twenty five μl of ddH_2_O was added to each well and a further 3 min vacuum was applied. Twenty μl of ddH_2_O was added to each well; the plate covered and vortexed at 1,000 rpm for 10 min. The remaining contents of each well (clean PCR product) was then transferred for storage. The Big Dye terminator cycle sequencing reaction was then performed using a 10-µl reaction mixture containing 0.5 µl of BigDye v3.1 Applied Biosystems™ (ABI Ltd, Warrington, Cheshire. UK), 2 µl of PCR product, 1.5 µl of 5 picomolar (pM) forward or reverse primer, 3.5 µl of ddH_2_O, and 2.5 µl of sequencing buffer (ABI Ltd) containing 5x Tris-HCl and MgCl_2_. The sequencing reaction mixture was purified using the Montage sequence cleanup plate (Millipore, Watford, UK): 25 μl of injection solution (Millipore,) were added to each well, and the total 35 μl was transferred to the blue plate and vacuumed for 3 min. A further 25 μl of injection solution was added to each well and vacuumed again for 4 min. Twenty five μl of injection solution was again added to each well, the plate covered and vortexed for 10 min. The remaining volume was transferred to the sequencing plate and run on the ABI 3700 sequencer. The DNA sequence was analyzed using DNAStar® Inc. (Madison, WI). To check segregation of the mutant alleles with disease, restriction digests were performed. Family 1 was investigated using HaeIII enzyme (Figure 2; New England Biolabs Ltd, NEB, Hitchin, Herts, UK), while in family 2 a digest using an *E. coli* strain that carries the *BglI* gene from *Bacillus globigii* (Bg*l*I [NEB]) was performed. One unit (0.2 μl) of enzyme was combined with 2 μl of 1x NEBuffer 3 (NEB, Herts, UK), 10 μl of PCR product and brought to a 20 μl reaction volume with deionized H2O (ddH_2_O) and digested for 16 h at 37 °C. Heat inactivation was performed at 80 °C for 20 min and the product run out on a 5% agarose gel.

### Clinical examination

Affected individuals underwent a full clinical examination, including Logarithm of the Minimum Angle of Resolution (LogMAR) visual acuity, color vision testing using the Ishihara plates, refraction, slit-lamp biomicroscopy, Goldmann visual field testing, and color fundus photography. A subset of patients underwent electroretinography using International Society for Clinical Electrophysiology of Vision (ISCEV) protocols [6,7].

## Results

In family I, analysis of the data from the Affymetrix gene chip array revealed a region of 47 contiguous homozygous single nucleotide polymorphisms (SNPs) on chromosome 10q23.1–23.3 spanning an interval of 10 cM in all four affected individuals. The parents and two unaffected siblings were heterozygous for these SNPs. This was the only significant region where affected individuals were homozygous and unaffected family members were not. The region contained approximately 45 genes of which four, *PCDH21* (OMIM 609502); *LRRC21* (leucine-rich repeat-containing protein 21 precursor, retina-specific protein photoreceptor-associated LRR superfamily [8]); *RGR* (retinal pigment epithelium [RPE]-retinal G protein-coupled receptor - OMIM 600342), and *OPN4* (melanopsin - OMIM 606665), were highly expressed in the retina relative to other human tissues (NCBI).

**Figure 3:**
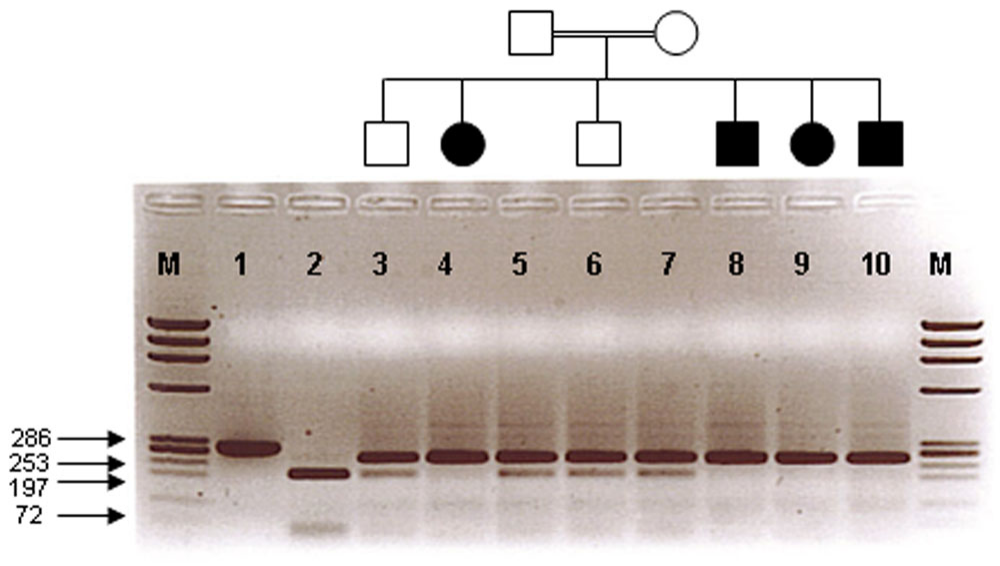
Co-segregation study of members of family 1 using the HaeIII restriction enzyme. Lane 1 is an undigested PCR product (286 bp). Lane 2 is a completely digested PCR product representing the wild-type allele (two products 197 and 56 bp). Affected individuals (lanes 4, 8, 9, and 10) show the homozygous mutant allele (one product 253 bp). Unaffected individuals, carriers (lanes 3, 5, 6, and 7) display the mutant allele in a heterozygous state (two products 253 and 197 bp). M represents the ϕX174RF DNA HaeIII marker.

Direct sequencing of the coding regions and intron–exon boundaries of *LRRC21* and *RGR* showed no variants in affected individuals. Sequencing of the 17 coding exons of *PCDH21* showed a homozygous single base deletion within exon 4, c.338delG p.G113AfsX1 (Figure 2A). The mutation segregated as a recessive allele in the family (Figure 3) and was not detected in 292 control chromosomes (86 UK blood donors, European Collection of Cell Cultures (ECACC) and 60 ethnically matched control individuals). In addition, the specific change was not found in a panel of DNA from 192 arRP and 96 Leber congenital amaurosis (LCA) patients recruited from Moorfields Eye Hospital (London, UK).

**Figure 4:**
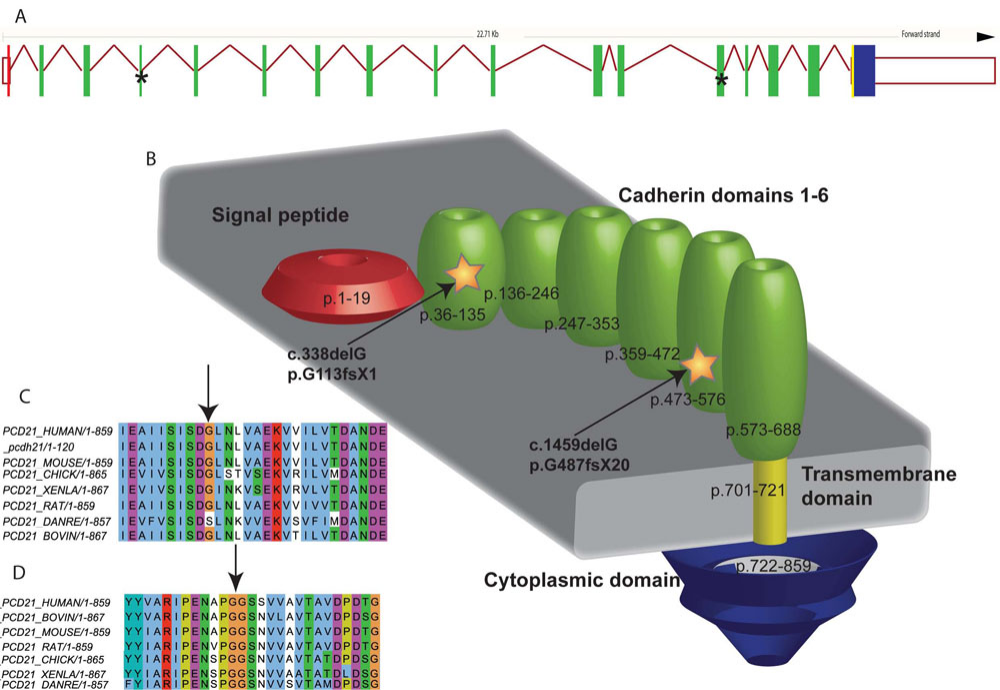
These are illustrations of both the exon structure and a schematic of the Protocadherin-21 (PCDH21) protein with indications of the location of the two deletions. Sequence alignments demonstrate the conservation of both amino-acid positions across several species. Panel **A**: PCDH21 exon structure as derived from ensembl: the exons are colored according to domain structure, and, as illustrated in Panel **B**, with asterisks to denote the location of the novel deletions. Panel **B** is a schematic of the cellular domain structure derived from UniProt (created using Adobe Illustrator-Adobe Systems Inc.) with the protein numbers corresponding to each of the structural domains. Panel **C** is an sequence alignment created using PipeAlign for PCDH21 mutation p.G113fsX1, that was identified in family 1, and shows that there is a high level of conservation at this amino acid position-except for species *Danio rerio*. Panel **D** is a further alignment around the site of the PCDH21 deletion p.G487fsX20, identified in family 2, demonstrating a high level of conservation at this amino acid position and therefore providing evidence of the likely deleterious effect of this variant.

In family 2, the largest region of autozygosity, which contained 195 contiguous homozygous SNPs, was on chromosome 10q. Direct sequencing of *PCDH21* revealed a homozygous single-base deletion in exon 13, c.1459delG, p.G487GfsX20 (Figure 2B). This is predicted to lead to truncation of the protein through a premature stop codon, 19 codons downstream of the deletion. This deletion segregated as a recessive allele in family 2 and was not found in 292 control chromosomes or in our LCA and arRP panel. Both deletions reside within the cadherin domains of the protein and occur at amino-acid positions that are highly conserved across species (Figure 4A-D).

**Figure 5:**
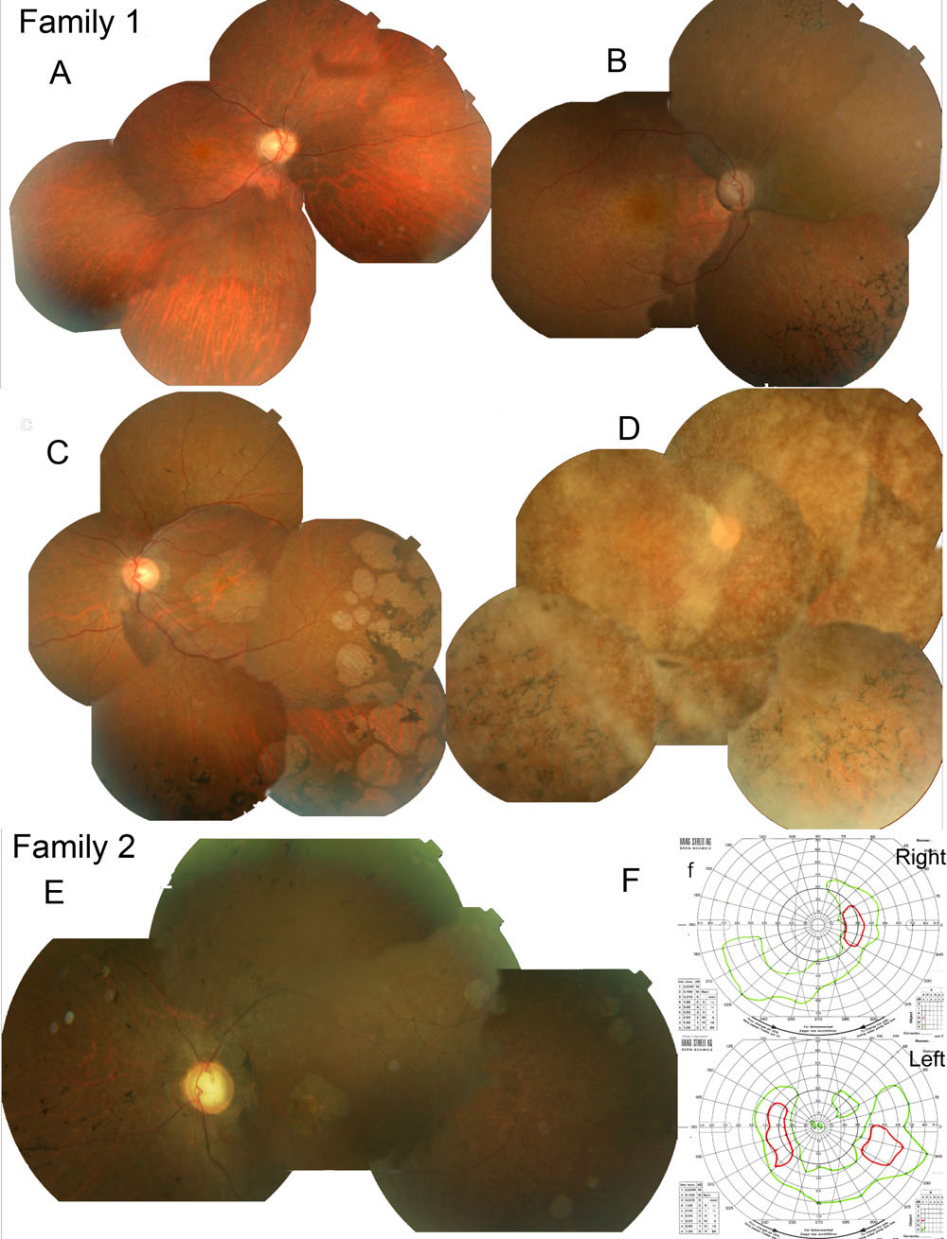
Color fundus composite photographs of affected individuals from families 1 and 2 and Goldmann visual fields from the proband in family 2. Panel **A** shows a fundus composite of the right eye from 34-year-old male (IV:6) from family 1; his visual acuity (VA) was 0.1 LogMAR with an electroretinogram (ERG) that was consistent with a “mild cone–rod dystrophy.” The image shows evidence of vessel attenuation and early peripheral retinal atrophy with few bone spicules. Panel **B** shows a fundus composite of the right eye in 36-year-old female (IV:5) from family 1 whose VA was 0.3 LogMAR, and who had absent color vision; her ERG was consistent with a diagnosis of retinitis pigmentosa. The fundus image shows macular depigmentation, dense peripheral bone spicules, and vessel attenuation. Panel **C** displays the color fundus composite from the left eye of 42-year-old male (IV:3) from family 1. His VA was 1.3 LogMAR, and the image displays circular patches of RPE atrophy both at the macula and in the periphery with associated peripheral pigment migration. His ERG was described as consisted with “advanced RP.” Panel **D** shows a color fundus composite from the right eye of 44-year-old female (IV:2) from family 1; Her visual acuity was limited to Counting Fingers (CF) and the view of the posterior pole is obscured by dense asteroid hyalosis as seen; there is peripheral bone spicule pigmentation and circular pigment epithelial atrophy was observed in the far periphery. Panel **E** shows the left eye color fundus composite of 46-year-old male (II:1) from family 2. His VA was recorded as Hand Movements (HM) at his most recent examination. No color vision was ever detected; the fundus image shows an atrophic retina, with vessel attenuation, RPE atrophy around the disc, at the macula and in the periphery, and associated peripheral pigment migration. His ERG when tested at age 32 years was undetectable. Panel **F** displays the Goldmann visual fields for patient II:1 with the V4e isopter in green and III4e in red: there are peripheral islands of residual vision and no response centrally to the largest and brightest target (V4e) in the right eye and small areas of response in the left eye.

The clinical history and phenotype in affected members of both families was similar. In family 1, nyctalopia was first reported in late teenage years, and photophobia occurred in the mid-twenties. The visual acuity was 0.1 (LogMAR) in the early 30s, deteriorating to “hand movements” by the early 40s. All affected family members had a severe color vision defect, and all had a low myopic refractive error. Fundus examination revealed vessel attenuation, diffuse retinal pigment epithelial changes, and sparse bone spicule pigment migration in the retinal periphery in the younger patients. With disease progression, there was dense pigment migration and atrophy both at the macula and in the periphery (Figure 5A–D). Electroretinography (ERG) in the three oldest affected individuals (age 42, 40, 34) showed both rod and cone responses; the ERG in the youngest affected family member, age 32 years (IV6), showed a cone–rod pattern of disease.

Only one affected individual in family 2 was available for examination. He had onset of symptomatic night blindness at 18 years of age. When examined at 30, his visual acuity was 0.2 LogMAR in each eye, but this had declined to 1.5 LogMAR in each eye at the age of 44 years and hand movements (HM) by the age of 46 years. His refraction measured −5 diopters spherical equivalent. Color vision was very abnormal at his initial examination. His fundus findings were of early RPE depigmentation at the macula at age 32 years. Later there were circular patches of pigment epithelial atrophy both at the macula and in the periphery associated with pigment migration and vessel attenuation (Figure 5E). Goldmann visual field testing at 46 years of age revealed residual peripheral islands of vision to the largest and brightest targets (V4e and III4e; see Figure 5F). The ERG at age 34 years revealed no detectable rod or cone responses in either eye.

## Discussion

PCDH21 is photoreceptor-specific cadherin involved in the assembly of outer segment discs [2]. The protein is present at the base of the developing outer segment and co-localizes with Prominin 1 – a gene encoding a pentaspan transmembrane glycoprotein (PROM1). Both proteins are involved in disc morphogenesis [9]. Mutations in *PROM1* have been implicated in autosomal dominant cone rod dystrophy and arRP [10,11]. Yang et al. [9] postulated that PCDH21, PROM1, and cytoskeletal actin are responsible for the formation of nascent discs at the base of the outer segment model whereby new disc membranes form by an evagination of the ciliary plasma membrane followed by rim growth. In contrast to the *pcdh2 ^−/−^* mice generated by Rattner et al. [2] in which the outer segments were shortened, fragmented, and degenerate, the mutant *prom1* (R373C) transgenic mice have stacks of disc membranes that were of excessive size and aligned perpendicular to normal disc orientation.

*PCDH21* is a good candidate for human inherited retinal degenerations, and Bolz et al. [5] screened a large number of arRP, LCA, and Usher syndrome type 1 patients; three heterozygous variants were described but none that were shown to be definitely disease associated. Our study provides evidence that *PCDH21* is a rare cause of nonsyndromic RP.

The retinal phenotype is consistent in the two families with night vision loss in the late teenage years but relatively well preserved central vision until the late 30s. In the early 40s vision deteriorates markedly. There are characteristic circular patches of pigment epithelial atrophy both at the macula and in the periphery appearing late in the disease process. This atrophy of the RPE and inner choroid occurring together with photoreceptor dysfunction suggest that phagocytosis of abnormal outer segments of the mutant retina might have a toxic effect on the RPE. All affected individuals examined had severe color vision defects, and ERGs performed at the referring institution (family 1) and at our institution (family 2) revealed severe cone and rod degeneration; the youngest affected individual had a cone–rod pattern of disease, suggesting that the earliest manifestation of mutations in *PCDH21*is a cone–rod dystrophy.

In summary, we have identified two consanguineous families who have separate, novel, homozygous, frame-shifting deletions that result in stop mutations that would be expected to abrogate protein function and cause nonsense-mediated decay [12] of the transcribed RNA. The phenotype is of a retinal dystrophy that begins in late teenage years with nyctalopia and visual field defects and by the fourth decade results in significantly impaired central vision.
